# What happens to food choices when a gluten-free diet is required? A prospective longitudinal population-based study among Swedish adolescent with coeliac disease and their peers

**DOI:** 10.1017/jns.2013.24

**Published:** 2014-02-13

**Authors:** E. Kautto, P. J. Rydén, A. Ivarsson, C. Olsson, F. Norström, L. Högberg, A. Carlsson, L. Hagfors, A. Hörnell

**Affiliations:** 1 Department of Food and Nutrition, Umeå University, Umeå, Sweden; 2 Umeå School of Gender Studies, Umeå University, Umeå, Sweden; 3 Department of Public Health and Clinical Medicine, Epidemiology and Global Health, Umeå University, Umeå, Sweden; 4 Division of Paediatrics, Department of Clinical and Experimental Medicine, Faculty of Health Science, Linköping University, Linköping, Sweden; 5 Department of Paediatrics in Norrköping, County Council of Östergötland, Norrköping, Sweden; 6 Department of Pediatrics, Lund University, Lund, Sweden

**Keywords:** Coeliac disease, Gluten-free diets, Food choices, Screening, CD, coeliac disease, ETICS, Exploring the Iceberg of Celiacs in Sweden, EU, European Union, FIL, food intake level, GF, gluten free, PAL, physical activity level

## Abstract

A dietary survey was performed during a large screening study in Sweden among 13-year-old adolescents. The aim was to study how the intake of food groups was affected by a screening-detected diagnosis of coeliac disease (CD) and its gluten-free (GF) treatment. Food intake was reported using a FFQ, and intake reported by the adolescents who were diagnosed with CD was compared with the intake of two same-aged referent groups: (i) adolescents diagnosed with CD prior to screening; and (ii) adolescents without CD. The food intake groups were measured at baseline before the screening-detected cases were aware of their CD, and 12–18 months later. The results showed that food intakes were affected by screen-detected CD and its dietary treatment. Many flour-based foods were reduced such as pizza, fish fingers and pastries. The results also indicated that bread intake was lower before the screened diagnosis compared with the other studied groups, but increased afterwards. Specially manufactured GF products (for example, pasta and bread) were frequently used in the screened CD group after changing to a GF diet. The present results suggest that changing to a GF diet reduces the intake of some popular foods, and the ingredients on the plate are altered, but this do not necessarily include a change of food groups. The availability of manufactured GF replacement products makes it possible for adolescents to keep many of their old food habits when diagnosed with CD in Sweden.

In Sweden, as well as in many other countries, the gluten-containing grains wheat, rye and barley constitute the main ingredient of staple foods such as bread and pasta^(^^1^^)^. A diagnosis of coeliac disease (CD) requires a dietary treatment where gluten is eliminated, as in susceptible individuals it promotes an inflammatory reaction in the small intestine causing villous atrophy^(^^2^^)^. The gluten-free (GF) dietary treatment should be strict, life-long and is facilitated by an acceptance of GF food appearance, taste and texture^(^^3^^)^. However, durable food changes have in the literature been shown to be hard to accomplish no matter the circumstances^(^^4^^,^^5^^)^, and staple foods are particularly hard to abandon because they are so closely connected with customs and culture^(^^6^^)^. The selection of GF products in food stores has increased, which should facilitate the transition to GF food^(^^1^^)^. Home-baking also increases the repertoire of GF products^(^^7^^)^. GF mixes are used as alternatives to ordinary flour when baking or preparing different dishes at home. Since GF mixes have different baking properties compared with ordinary flour^(^^8^^,^^9^^)^, some new knowledge and skills will be needed when baking.

Specially manufactured GF replacement products are usually more expensive than ordinary staple food. In Sweden, children with CD below 16 years of age are therefore supported by the governmental health care system by subsidised prescription of GF products^(^^10^^)^. The dietary treatment is almost always effective and promotes health; however, the requirement of following a strict GF diet sometimes is experienced as socially stigmatising^(^^11^^)^. To our knowledge, changes in food choices after being prescribed a GF diet has not been explored previously; neither after CD diagnosis due to health problems nor after screening.

Many studies describe adolescents' GF diet as high in SFA and sugars, with a low intake of fibre and some micronutrients^(^^12^^,^^13^^)^. In a previous study we confirmed this finding; however, importantly, the dietary intake of a same-aged non-coeliac reference group had the same natural drawbacks^(^^14^^)^.

The aim of the present study was to explore how CD diagnosed by screening in early adolescence affected overall food choices in comparison with adolescents diagnosed with CD at an earlier age and a reference group of adolescents without CD both with similar age and sex distribution. Our hypothesis was that the CD diagnosis followed by prescription of a GF diet would result in a reduced consumption of bread, pasta and other flour-containing food and an increase of naturally free GF products such as rice and potatoes. Nutritional intake is not considered in the present study.

## Subjects and methods

### Study design

The study ETICS (Exploring the Iceberg of Celiacs in Sweden) involved all adolescents in five Swedish cities (and surroundings) in 2005–2006 in a CD screening. After informed consent, weight and height were measured, blood samples were taken, and later adolescents and their parents filled out questionnaires. The study is described elsewhere in detail^(^^15^^)^.

In addition, a sample of these adolescents was invited to a case-referent dietary study. This sub-study involved all CD cases, both newly screening-detected and those previously diagnosed, and a sample of their non-CD peers as comparison. Food choices were measured at baseline, before the screening-detected cases were aware of their CD, and 12–18 months later.

### Coeliac disease screening and ascertainment

All participants had their blood samples analysed for tissue transglutaminase antibodies of IgA-type (tTG-IgA), and some also for complementary diagnostic tests as described elsewhere^(^^15^^)^. Individuals with levels above a pre-set cut-off were offered a small-intestinal biopsy, which is the ‘gold standard’ for CD diagnosis. CD diagnosis required a small-intestinal mucosa with villus atrophy or borderline mucosa in combination with symptoms and/or signs compatible with CD. Previously diagnosed CD was reported by the parents and confirmed through the National Register for Celiac Disease and/or medical records. Thus, both CD groups were diagnosed according to diagnostic criteria published in 1990 by the European Society for Paediatric Gastroenterology, Hepatology and Nutrition (ESPGHAN)^(^^16^^)^.

Participants with tTG-IgA below the pre-set cut-off were unlikely to have CD and random samples of these were chosen as referents at baseline or follow-up.

### Subjects

Altogether, 10 041 adolescents were invited to the CD screening study and 7567 participated (75 %). Out of these, 192 had tissue transglutaminase IgA (tTGA) values above the cut-offs, and thus had suspected untreated CD. When the present sub-study was initiated the diagnosis of CD had been verified in 145 out of these. In addition sixty-two adolescents had CD that had been diagnosed prior to the screening. At baseline 1151 referents were included, which was about four times the number of verified suspected CD cases. Later the CD diagnosis was rejected in two adolescents – one from the screened group and one from the previously diagnosed group – resulting in 144 screened CD and sixty-one previously diagnosed CD.

**Fig. 1.**
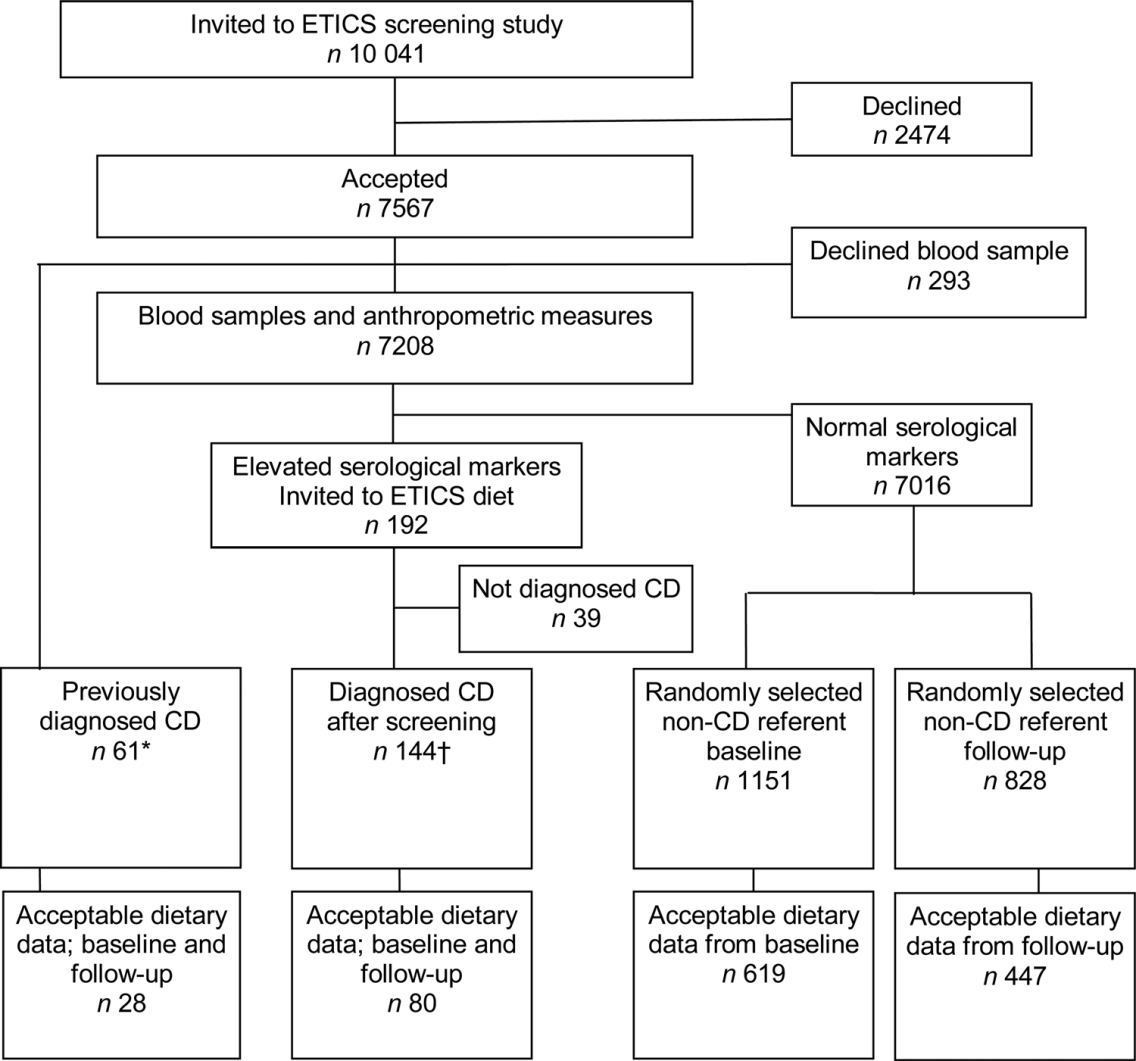
Flowchart of participants through the ETICS (Exploring the Iceberg of Celiacs in Sweden) diet study. CD, coeliac disease. * An additional five previously diagnosed CD cases were not included in this dietary study. † An additional nine CD cases were diagnosed in the ETICS study after the start of the follow-up study and, therefore, they were not included in the invitation to the dietary study.

Additional inclusion criteria were complete information on weight and height as well as reasonably answered FFQ with requirements given below. Thereafter the screening-detected CD group consisted of eighty cases and the previously diagnosed CD group of twenty-eight cases (Fig. 1). The referent group at baseline and follow-up consisted of 619 and 447 referents, respectively. Age and sex distribution for each group is given in Table 1. The previously diagnosed CD group was diagnosed at the median age of 1·5 (interquartile range 1·8–8·2) years.

**Table 1.** Characteristics of participants in the ETICS (Exploring the Iceberg of Celiacs in Sweden) diet study (Mean values and standard deviations, and median values and 25th–75th percentiles)

	Screened CD				Previously diagnosed CD				Non-CD referent					
	Baseline (*n* 80)		Follow-up (*n* 80)		Baseline (*n* 28)		Follow-up (*n* 28)		Baseline (*n* 619)		Follow-up (*n* 447)		*P*†	
	Mean	sd	Mean	sd	Mean	sd	Mean	sd	Mean	sd	Mean	sd	Baseline	Follow-up
Age (years)	13·1	0·3	14·6	0·3	13·3	0·4	14·5	0·2	13·2	0·4	14·6	0·4	0·115‡	0·328‡
Median	13·1		14·6		13·3		14·6		13·2		14·6			
25th–75th percentiles	12·9–13·3		14·4–14·7		13·0–13·6		14·4–14·7		12·9–13·4		14·3–14·8			
Sex, girls (%)	49		49		61		61		54		58		0·499§	0·283§
Height, *z*-score║	0·4	1·0			0·3	1·0			0·5	1·0	0·5	1·0	0·457‡	
Median	0·3				0·2				0·5		0·6			
25th–75th percentiles	–0·3 to 1·1				–0·6 to 0·9				–0·1 to 1·2		–0·3 to 1·1			
Weight, *z*-score║	0·2	1·0			0·1	0·9			0·3	0·9	0·3¶	0·9	0·537††	
Median	0·2				–0·1				0·3		0·3¶			
25th–75th percentiles	–0·3 to 0·8				–0·7 to 0·8				–0·4 to 0·9		–0·3 to 0·9			
BMI (kg/m^2^), *z*-score║	–0·01	1·1			–0·1	0·9			0·1	1·0	0·1¶	0·9	0·740††	
Median	0·02				–0·3				0·1		0·1¶			
25th–75th percentiles	–0·5 to 0·6				–0·8 to 0·6				–0·6 to 0·73		–0·5 to 0·7			
ISO-BMI > 25 kg/m^2^ (%)║	15				14				16		16¶		0·921§	
PAL‡‡			1·8	0·15			1·8	0·1			1·79	0·15		0·694‡
Median			1·8				1·8				1·75			
25th–75th percentiles			1·7–1·9				1·7–1·9				1·67–1·88			
FIL (EI/BMR)	1·6	0·4	1·6	0·5	1·7	0·5	1·6	0·4	1·6	0·42	1·54	0·44	0·340‡	0·730‡
Median	1·5		1·5		1·7		1·5		1·5*		1·5*			
25th–75th percentiles	1·3–1·9		1·2–1·9		1·3–2·0		1·3–1·9		1·3–1·9		1·2–1·8			
Energy reporting (*n*)§§														
Low	17		26		4		7		107		158		0·720§	0·556§
Acceptable	58		49		22		20		472*		265*			
High	5		5		2		1		40		24			

CD, coeliac disease; ISO-BMI, BMI for children; PAL, physical activity level; FIL, food intake level; EI, energy intake.

* *P* < 0·05.

† Between the three groups.

‡ Calculated by ANOVA.

§ Calculated by χ^2^ test.

¶ *n* 446.

†† Calculated by Kruskal–Wallis test.

║ Calculated from measured height and weight at admission to ETICS for all participants.

‡‡ Measured at ETICS diet at follow-up.

§§ Assessed by means of Goldberg's cut-off^(17,18)^.

### Anthropometrics

The participants' weight and height were measured using standard procedures by the school nurse at admission to the ETICS project (Table 1). A total of sixteen individuals lacked information about weight at baseline, thus not fulfilling the inclusion criteria. The weights and heights of the screened CD group were repeatedly measured in hospitals during follow-up visits. Due to the lack of follow-up weight records for the other three groups – the previously diagnosed CD group and the non-CD groups – their weight-for-age at the time of the dietary measurement was estimated using Epi Info^TM(^^17^^)^. In Epi Info, weight measurements at the time of admission to ETICS were converted to z-scores for age and sex based on growth charts from the Centers for Disease Control and Prevention, USA. New weights were calculated based on the assumption that the adolescents did not deviate from their original *z*-score (Table 1). BMI (kg/m^2^) was calculated, and converted to normal weight, overweight and obese by sex and age according to Cole's definition^(^^18^^)^ (Table 1).

### Dietary assessment

The adolescents and their parents were encouraged to jointly complete the FFQ. The baseline FFQ was sent to their home from June to November 2006, which was on average 7 (range 2–16) months after admission to the project. The FFQ were returned fairly similarly between the seasons but with a peak during the summer months of June (34 %) and July (22 %). The follow-up FFQ was sent out 12–18 months after baseline, equally distributed over the year between the screened CD, previously diagnosed CD, and non-CD follow-up group. Most were returned during the winter months of December (45 %) and January (18 %) and none during the summer. In the present study an inclusion criterion for individuals in the CD groups was a returned FFQ at baseline as well as at follow-up.

In brief, the FFQ was developed especially for the ETICS diet study and pre-tested among adolescents of the same age. The main focus of the FFQ was to study the overall food intake highlighting (normally) gluten-containing products. The FFQ covered the overall food intake of the preceding 4-week period. It included fifty-seven food items, such as fruits, vegetables, bread, fats, pastries, cold cuts, milk and yogurt, potatoes, rice, pasta, meat and meat products, chicken, fish, traditional dishes and ‘discretionary calories’. Questions such as what type of bread, fat, milk and yogurt were included. At baseline, the GF dietary treatment had already been prescribed for the previously diagnosed CD group and at follow-up both CD groups had been prescribed a GF diet.

Compliance with the GF diet was self-reported through an overall question in the beginning of the FFQ and as separate questions about how often each normally gluten-containing food item was GF. For the separate food items, the answer alternatives ‘never’/‘sometimes’/‘mostly’/‘always’ were converted into the proportions 0, 25, 75 and 100 % of the reported frequency when calculating amounts of the different foods. The FFQ was semi-quantitative and portion sizes were reported by comparing the amount eaten with photographs of different portion sizes in a photographic booklet published by the Swedish National Food Administration (NFA)^(^^19^^)^, using household measures or natural sizes (for example, one apple). Frequency of dietary intake was reported on a six-level scale from ‘never in the 4 weeks’ to ‘two or more times daily’ for most food items. For milk and yogurt the upper limit was ‘five or more times daily’, and bread was reported as number of slices or pieces per d.

At baseline, a total of eighty-eight FFQ did not meet inclusion criteria of being reasonably answered, forty-four due to missing amounts on more than four food items reported eaten with a frequency of 1–3 times per 4 weeks, and forty-four due to missing amounts for more than three food items with a reported maximum frequency of once per d. For the follow-up FFQ, the participants were telephoned and asked about missing or implausible data; therefore amounts should be given for all food items to be included in the analysis.

The food intake reported as frequencies and quantities were converted to g/d by multiplying frequency of intake by the estimated portion size in grams. The energy and nutritional content was calculated using the software program Dietist XP 3·1 (Kost och Näringsdata), based on the Swedish National Food Administration (NFA) database (version 2010-03-15) of the nutritional content in the food. Food items were grouped in order to facilitate the analysis of dietary changes with a special interest in gluten-containing food items and their GF counterparts (Table 2). The food groups were divided in two main groups: (1) grain containing; and (2) non-grain containing. Pastries were placed under grain products when counted separately and also under non-grain products when included in ‘discretionary calories’ (which include sweets, snacks, ice-cream, pastries, soft drinks, jam and sweet dessert soups) (Table 2). To enable comparisons between individuals regardless of their energy intake, all dietary intakes were converted to intake per 4184 kJ (1000 kcal) as is common practice.

**Table 2.** Definition of food groups in the ETICS (Exploring the Iceberg of Celiacs in Sweden) diet study

Food group	Description
Grain products	Both gluten-containing and gluten-free
Bread, total	Soft and crisp
Bread, low fibre	Low fibre
Bread, whole grain	Soft bread labelled with the Swedish ‘Keyhole’ symbol indicating high fibre or wholegrain content
Crisp bread	
Cereals, total	Products where gluten-containing cereals constitute a main ingredient and their gluten-free substitutes
Breakfast cereals	Breakfast cereals, porridge
Pastries	Cookies, cake, buns
Pasta	Pasta, couscous, bulgur, pearl barley
Pizza	
Pancakes	
Fish fingers	Breaded fish
Chicken nuggets	Breaded chicken
Non-grain products	
Fruit	Fruits, berries, dried fruits
Vegetables	Vegetables, root vegetables, not potatoes
Potatoes	Potatoes, French fries, not potato crisps
Dairy products	Milk, milk products, cheese
Fat as spread	Butter, margarine
Eggs	
Meat, total	Meat, processed meat products (i.e. sausages, meatballs, cold cuts)
Meat, processed	Processed meat products (i.e. sausages, meatballs, cold cuts)
Fish, total	Fish, shellfish, processed fish products
Poultry, total	Poultry, processed poultry products (i.e. sausages, meatballs, cold cuts)
Rice	
‘Discretionary calories’	Sweets, snacks, ice cream, pastries, soft drinks, jam, dessert soups

### Quality of the food records

A total food intake level (FIL) was estimated for each participant, in order to detect and exclude individuals who are unrealistic energy reporters. FIL was estimated by reported energy intake divided by the estimated BMR^(^^20^^,^^21^^)^. A FIL value below the 5th percentile (at baseline <0·9 for both boys and girls; at follow-up <0·9 for boys and <0·8 for girls) or above the 95th percentile (at baseline >2·9 for boys and >2·6 for girls; at follow-up >2·7 for boys and >2·6 for girls) was deemed unrealistic and these adolescents were excluded from further analysis (Table 1).

At follow-up, questions about physical activity at school and during leisure time were added enabling estimation of physical activity level (PAL) for each participant. The number of hours of physical activity during weekdays and weekends were converted into average hours per d. Activities reported as making you slightly breathless and warm were classified as ‘light’ and given a metabolic equivalent (MET) value of 4·2, while activities giving a high pulse, breathlessness and sweatiness was classified as ‘strenuous’ with a MET value of 8·4.

The Goldberg cut-off method was used to identify low energy reporters, acceptable energy reporters and high energy reporters^(^^21^^,^^22^^)^. The calculated cut-offs based on the PAL indicate whether FIL is plausible or not. The lower and upper cut-offs were calculated uniquely for each individual at follow-up based on their PAL. At baseline information about the adolescents' physical activity was missing, therefore the mean PAL value for the total group of participants measured at follow-up was used, adding a margin of safety (±1 sd). A PAL value of 1·6 was used to calculate the lower FIL cut-off and 1·9 to calculate the upper FIL cut-off for all participants. At baseline 76 % were classified as adequate reporters and at follow-up 60 %. Subjects with a FIL below the lower cut-off were classified as low energy reporters and those above the upper cut-off as high energy reporters, in the present paper jointly called mis-reporters (Table 1). As exclusion of these mis-reporters did not notably alter the results, i.e. the order of the groups remained although the significance levels attenuated, all participants were included in the final results.

### Statistical analysis

Both proportions of participants reporting eating a specific food group and the amounts eaten at baseline and follow-up were studied. The Kruskal–Wallis test was used to analyse differences between groups at baseline and at follow-up. The Mann–Whitney *U* test was used to analyse differences between the non-CD referent groups and differences between screened CD, previously diagnosed CD and non-CD at baseline and follow-up, respectively. Wilcoxon's signed rank test was used to analyse changes in food intake between baseline and follow-up in the screened CD and previously diagnosed CD groups, respectively.

Due to the many tests performed and the differences in group sizes, effect sizes were calculated for each change/difference in order to determine if they were of practical and/or theoretical use^(^^23^^)^. For the Mann–Whitney *U* test the *z*-value was used to calculate the effect size as an approximation value of *r* (*r* = *z*/square root of *N* (*N* = total number of cases)) and the effect size of a Wilcoxon signed rank test was performed (*r* = *z*/square root of *N* (*N* = total number of cases × 2)). Interpretation was carried out according to Cohen's definition (1988), i.e. differences of 0·1 were considered a small effect (S), 0·3 a medium effect (M) and 0·5 a large effect (L)^(^^24^^)^. Level of significance was set at *P* < 0·05.

### Ethical considerations

The present study was conducted according to the guidelines laid down in the Declaration of Helsinki and all procedures involving human subjects/patients were approved by the Regional Ethical Review Board in Umeå (no. 04–156M). Written informed consent was obtained from all subjects. The participants could at any time discontinue their participation. Each individual was assigned a temporary code replacing their personal identity number, and security for the database was high with access only available to key researchers.

## Results

### Self-reported compliance to a gluten-free diet

At follow-up, all but nine of eighty participants in the screened CD group reported that they always followed a GF diet; six of these reported that they mostly followed a GF diet and three stated that they did not follow a GF diet at all. In the previously diagnosed CD group all but one of twenty-eight reported that they always followed a GF diet, the one participant who did not, reported that the diet was mostly GF.

### Reported overall food intake: comparisons between groups

At baseline, the screened CD group and the non-CD referent group reported a very similar intake of most food groups except for bread and crisp bread, of which the screened CD group reported a lower intake (Table 3). The previously diagnosed CD group reported a deviating intake of several food groups compared with the other two groups at baseline, with the highest intake of bread and fat as spread, and the lowest intake of pastries, pizza, total poultry and chicken nuggets. The analysis revealed that observed differences were mostly of small effect size.

**Table 3.** Intake (g/4·2 MJ) by food groups for the screened coeliac disease (CD) cases, previously diagnosed CD cases and the non-CD referent group at baseline and follow-up, respectively (Median values and and 25th–75th percentiles)

	Baseline									Follow-up									
	Screened CD (*n* 80)		Previously diagnosed CD (*n* 28)		Non-CD baseline (*n* 619)		*P* value and effect size			Screened CD (*n* 80)		Previously diagnosed CD (*n* 28)		Non-CD follow-up (*n* 447)		*P* value and effect size			
	Median	25th–75th percentile	Median	25th–75th percentile	Median	25th–75th percentile	*	†	‡	Median	25th–75th percentile	Median	25th–75th percentile	Median	25th–75th percentile	*	†	‡	§
Grain products																			
Bread, total	41·0	26·3–54·6	50·9	33·6–79·7	47·9	32·4–66·2	0·023	0·020	0·014	41·0	29·6–53·8	61·3	38·0–74·0	46·7	29·6–63·6	0·041	0·009	NS	NS
								S║	S								M¶	S	VS**
Bread, low fibre	20·1	6·4–32·1	23·4	1·7–41·7	21·3	0·0–41·0	NS	NS	NS	14·4	4·6–24·4	19·5	5·4–46·8	18·1	0·0–39·1	NS	NS	NS	0·025
								S	VS								S	VS	S
Bread, whole grain	13·3	0·0–31·6	22·0	4·8–35·0	15·7	0·0–36·1	NS	NS	NS	20·1	8·3–36·7	22·0	2·0–43·5	16·0	0·0–35·7	NS	NS	NS	0·021
								S	VS								VS	S	S
Crisp bread	0·6	0·0–3·9	1·6	0·0–5·7	1·4	0·0–5·2	NS	NS	0·041	1·4	0·0–4·9	0·7	0·0–3·7	1·5	0·0–6·2	NS	NS	NS	NS
								S	S								S	VS	S
Cereals, total	150·6	121–177	128	100–164	148	124–177	NS	NS	NS	137	109–158	126	105–157	145	120–178	0·008	NS	0·013	0·014
								S	VS								VS	S	S
Breakfast cereals	25·9	12·0–55·4	46·0	19·3–109	26·3	12·4–51·8	NS	NS	0·048	29·0	15·1–55·9	25·8	10·5–47·2	30·6	15·0–56·6	NS	NS	NS	NS
								S	VS								S	VS	VS
Pastries	8·0	4·1–13·1	3·7	1·6–7·8	6·7	2·9–10·4	0·001	<0·001	NS	1·3	0·5–3·0	1·1	0·6–2·5	6·0	2·3–9·9	<0·001	NS	<0·001	<0·001
								M	S								VS	M	L††
Pasta	23·5	14·4–36·1	21·4	13·5–25·7	23·9	16·2–38·7	NS	NS	NS	19·8	14·5–33·2	22·2	13·5–27·2	25·0	16·6–37·2	0·025	NS	0·033	NS
								S	VS								VS	S	S
Pizza	13·6	7·7–20·0	0·7	0·0–17·5	14·3	8·6–20·5	0·010	0·016	NS	8·7	1·4–11·5	9·2	0·0–13·2	14·7	9·2–19·7	<0·001	NS	<0·001	<0·001
								S	S								VS	M	M
Fish fingers	1·6	0·0–3·4	1·0	0·0–3·1	2·1	0·0–3·8	NS	0·042	NS	0·0	0·0–1·8	0·0	0·0–1·0	1·8	0·0–3·7	<0·001	NS	<0·001	0·002
								VS	VS								VS	S	S
Chicken nuggets	0·0	0·0–1·7	0·0	0·0–0·0	0·0	0·0–1·6	0·015	NS	NS	0·0	0·0–0·0	0·0	0·0–0·0	0·0	0·0–0·0	<0·001	NS	<0·002	<0·001
								M	VS								S	S	M
Vegetables	25·9	12·0–54·5	46·0	19·5–104	26·3	12·4–51·8	NS	0·032	NS	29·0	15·1–55·8	25·8	10·6–47·1	30·6	15·0–56·6	NS	NS	NS	NS
								S	VS								S	VS	VS
Non-grain products																			
Potatoes	68·2	48·3–98·4	61·9	47·5–94·9	62·3	39·2–91·1	NS	NS	NS	75·4	48·8–109	79·8	44·7–99·8	61·3	39·0–84·7	0·005	NS	0·002	NS
								S	S								S	S	VS
Fat spread	4·8	3·0–8·7	10·1	7·1–13·3	6·3	4·1–9·6	<0·001	<0·001	0·016	5·8	4·1–8·3	8·9	4·4–11·6	6·1	4·0–9·3	NS	0·040	NS	NS
								M	S								S	VS	S
Eggs	3·1	0–7·5	3·9	0·9–7·9	3·3	1·2–9·1	NS	NS	NS	3·7	2·0–10·3	3·2	0·9–9·8	3·6	1·6–9·1	NS	NS	NS	0·025
								S	VS								S	VS	S
Meat, total	64·2	46·0–85·0	54·1	47·6–79·2	60·2	45·3–81·0	NS	NS	NS	59·5	44·3–79·2	54·6	45·3–72·5	55·6	38·4–74·7	NS	NS	NS	NS
								S	VS								VS	S	S
Processed meat	17·5	10·2–32·0	25·7	14·0–32·1	21·6	13·5–32·0	NS	NS	NS	27·9	17·8–44·2	25·3	19·1–33·8	25·7	14·9–40·1	NS	NS	NS	0·001
								S	S								S	VS	M
Fish and shell fish	9·6	5·1–19·2	7·3	4·3–13·3	10·8	5·6–18·8	NS	NS	NS	10·5	4·8–20·2	6·8	2·5–14·3	14·1	6·7–23·3	0·001	NS	0·043	NS
								S	VS								S	S	S
Poultry, total	12·6	5·9–19·4	6·3	4·0–14·4	13·3	5·9–22·9	0·017	0·005	NS	11·6	5·5–18·8	6·9	3·8–15·3	12·2	5·2–20·3	NS	NS	NS	NS
								VS	M								S	VS	VS
Rice	17·6	8·9–21·9	12·2	6·3–23·1	14·5	6·0–23·0	NS	NS	NS	19·0	12·7–28·8	22·2	11·6–28·0	17·4	7·9–25·3	NS	NS	NS	0·038
								S	S								VS	S	S
‘Discretionary calories’	116	79·5–169	91·8	60·4–158	106	68·7–161	NS	NS	NS	88·2	55·4–113	103	43·2–144	90·6	58·8–129	NS	NS	NS	<0·001
								S	VS								S	S	M

VS, very small, S, small; M, medium; L, large.

* Differences analysed between screened CD, previously diagnosed CD and non-CD groups with Kruskal–Wallis test at baseline and follow-up, respectively.

† Differences between screened CD and previously diagnosed CD groups analysed with Mann–Whitney *U* test.

‡ Differences between screened CD and non-CD groups analysed with Mann–Whitney *U* test at baseline and follow-up, respectively.

§ Differences between screened CD reported intake from baseline to follow-up analysed with Wilcoxon signed rank test.

║ Small calculated effect size, indicating an effect size as >0·1 and <0·29.

¶ Medium calculated effect size, indicating an effect size as >0·3 and <0·49.

** Very small calculated effect size, indicating an effect size as < 0·1.

†† Large calculated effect size, indicating an effect size as >0·5^(19,20)^.

At follow-up, analysis of the three groups revealed a changed pattern. The two CD groups were now most similar, although the previously diagnosed CD group still reported the highest intake of bread (Table 3). The screened CD group reported at follow-up a lower median intake of bread but they increased their intake compared with baseline of total cereals, pastries, pasta, pizza, fish fingers, chicken nuggets, fish and shellfish and a higher intake of potatoes compared with the non-CD referent group (Table 3). The analysis revealed that observed differences were mostly of small effect size, but differences of pastries and pizza were of medium effect size.

### Proportions of reported eaters: comparisons at baseline and follow-up

To check if the participants' choices had altered within the groups, a comparison of individual participant choices was undertaken (data not shown). At baseline all but one of the eighty in the screened CD group reported eating bread and, at follow-up, all did, although amounts were relatively small compared with the other two groups. Eaters of low-fibre bread increased from sixty-five to seventy-six, whole grain bread from fifty-three to sixty-five and crisp bread eaters increased from fifty to fifty-eight out of the eighty. Of the four who did not eat low-fibre bread at follow-up, two had not eaten it at baseline either, while seven participants did not report any intake of wholegrain bread at either baseline or follow-up. In all, fourteen participants in the screened CD group refrained from eating crisp bread at both time points.

All twenty-eight participants in the previously diagnosed CD group reported that they ate bread at both baseline and follow-up; low fibre bread intake was reported by twenty-one at baseline and by all twenty-eight at follow-up. Intake of whole-fibre bread was reported by twenty-one participants at both baseline and follow-up. Crisp bread eaters decreased from twenty-one to seventeen.

All participants in both non-CD referent groups reported that they ate some kind of bread. Differences observed were that there were fewer in the non-CD referent group at follow-up who reported that they ate low-fibre bread and whole-fibre bread, but more who ate crisp bread compared with the non-CD referent group at baseline.

The proportions of participants reporting an intake of fish fingers and chicken nuggets decreased in both CD groups between baseline and follow-up. Eating fish fingers was reported by more than 50 % of participants in both CD groups and the non-CD referent group at baseline. At follow-up, the proportion of fish finger eaters in the screened CD and previously diagnosed CD groups was reduced to about 30 % in both groups (twenty-four of eighty and eight of twenty-eight participants, respectively). In the non-CD follow-up referent group, 56 % reported eating fish fingers. The reported proportion of eaters of chicken nuggets decreased in the screened CD group from 30 % to 9 % (from twenty-four to seven at follow-up). At both time points only one (but a different) participant in the previously diagnosed CD group reported eating chicken nuggets. The proportion of reported eaters of chicken nuggets in the two non-CD referent groups was almost the same at 27 and 24 %, respectively.

### Reported overall food intake – comparisons within groups

Significant changes in the reported quantity (g/d) of different food groups in the screened CD group during the study from baseline to follow-up were reductions in low-fibre bread, total cereals, pastries, pizza, fish fingers, chicken nuggets and ‘discretionary calories’; and increases of wholegrain bread, rice, eggs and processed meat (Table 3). The estimated effect size revealed that differences of pizza, chicken nuggets, processed meat and ‘discretionary calories’ were of medium effect size, and the reduction of pastries of large effect size. The previously diagnosed CD group reduced their intake of crisp bread, pastries, fish fingers, vegetables and fat spreads. Comparisons between the non-CD referent groups showed that the non-CD follow-up group had a lower intake of low-fibre bread, total meat and ‘discretionary calories’ and a higher intake of vegetables, processed meat, fish, shellfish and rice compared with the non-CD baseline group (data not shown).

## Discussion

The main finding in the present study was that a change to a GF diet after a diagnosis of CD affects food intake despite frequent use of manufactured GF replacement products. The result also indicates that the intake of bread is affected even before the diagnosis of CD. A reduced consumption of many energy-dense food groups was seen in the screened CD group after the change to a GF diet.

The present study focuses on food choices, although we acknowledge that there are many other important issues concerning food intake among adolescents with CD, such as nutrient intakes and social eating. To our knowledge there are no published data on participants reporting food intake both before and after a GF treatment has begun with an equally narrow range among adolescent participants.

At baseline, the screened CD group and the non-CD referent group reported a similar food intake except for the lower intake of total bread, crisp bread and breakfast cereals in the screened CD group. A previous qualitative study, with the same CD-screened adolescents, studying experiences of being screened, showed that many of them were unaware of symptoms before the diagnosis but after starting with the GF diet they felt an improvement in health and realised that they previously had had symptoms^(^^25^^)^. This may be explained by an unconscious decrease of bread before diagnosis due to the symptoms experienced when eating gluten-containing foods. At follow-up the screened CD group had altered their food intake, becoming more like the previously diagnosed CD group. Our hypothesis that a prescribed GF diet would result in a lower intake of bread was, according to the results in the present study, proven wrong. The previously diagnosed CD group reported the highest intake of bread compared with the other groups at both baseline and follow-up, and the screened CD group increased their bread intake after they begun with the GF diet.

At baseline, the screened CD group and the non-CD referent group were most alike, but the changes in food choices after diagnosis made the two CD groups the most alike at follow-up. Specially produced GF products (for example, pasta and bread) were frequently used in the screened CD group after the change to a GF diet. Forasmuch as the foods we eat are important to our identity, these specially produced GF replacement products probably gave adolescents with newly diagnosed CD the alternative to keep their habitual food choices and mitigate the change to a GF diet^(^^26^^,^^27^^)^. In the present study, changing from gluten-containing products to special GF replacement products within the same food group was not defined as a change. This suggests the possibility that individuals' experience of change in their food intake after the introduction of a GF diet is larger than that which is visible in the present study. When analysing changes in common gluten-containing products other than bread, such as pastries, pasta, fish fingers, chicken nuggets and pizza, the groups diagnosed with CD had a significantly lower intake than the non-CD referent group at follow-up, which is in accordance with our original hypothesis. Naturally GF products such as potatoes and rice were frequently used by all groups; the intake of rice increased after diagnosis of CD in the screened CD group although not the intake of potatoes.

A dilemma in the present study is the difference in group size. In order to avoid misinterpretations of the results and in order to deepen our understanding when analysing data, estimations of effect sizes were calculated. Having a large sample increases the risk of over-valuing observed significant differences where the importance of the differences could be quite trivial^(^^28^^)^. This occurred, for example, when we compared the differences of reported intake between the two non-CD referent groups (data not shown), and found many significant differences; however, the estimated effect size revealed that the relevance of these differences was mostly small. On the other hand, a calculated large effect size on non-significant differences in a small sample, such as the changes in the previously diagnosed CD group between baseline and follow-up, suggests a need for further research with a larger sample size^(^^28^^)^. We are aware of the small number of participants in the previously diagnosed CD group, consisting of only twenty-eight individuals, which could be considered a too-small sample for a dietary study. However, compared with other studies^(^^12^^,^^29^^,^^30^^)^, the age range of our participants is very narrow, which partly compensates for the relatively few participants.

Our specially designed FFQ included more specific questions about food usually containing gluten than it did for other foods. Therefore, reports for the intake of these food groups are likely to be more detailed. The FFQ consisted of a fixed food list and the risk of mis-reporting increases if these foods do not match the foods usually eaten by the subject since the questionnaire will not be able to accurately describe foods eaten in detail. As mis-reported dietary intakes may seriously bias results, the dietary data were assessed and FFQ from extreme reporters were excluded from all analyses. Furthermore, through the use of the Goldberg cut-off^(^^21^^)^ low and high energy reporters were identified. As expected, a large number of participants were identified as being mis-reporters and, to control for this, analyses were made both including and excluding them. This yielded similar results, thus reducing the risk of misleading conclusions.

The fact that the majority of participants returned the questionnaire during different seasons of the year at baseline and follow-up may also have affected the results. However, comparing the groups cross-sectionally at baseline and follow-up should not pose any problem since the FFQ was distributed evenly to the groups during the year. Another limitation of the present study is the focus on adolescents as one homogeneous group, which means that differences or similarities between the sexes have not been visualised, and this could be interesting for further analysis as many studies show different food preferences between the sexes.

In the literature strict adherence to the GF diet is described as a big challenge especially for adolescents^(^^31^^,^^32^^)^. In the present study, the participants in the CD groups self-reported their compliance to the diet and mostly reported a strict adherence. However, compliance is often hard to accomplish and the motivation for patients to remain strictly GF seems to be very individual^(^^32^^–^^34^^)^. In clinical practice it is important to be aware of the many factors that motivate individuals when they make their food choices. Besides the demands of a food-related disease, choices are affected by emotional, social and symbolic factors which in turn are influenced by family eating patterns, peers' attitudes, and identification with special food items^(^^35^^–^^37^^)^. Therefore a comprehensive perspective should be taken during counselling. The possibility of individually adjusting the dietary advice should be especially important for the dietitian to address when first introducing the GF diet to a newly diagnosed patient with CD. Adolescents diagnosed with CD may need extra guidance to discover GF alternatives facilitating exclusion of gluten from their diets. The dimension of how individuals experience this dietary treatment is not measured in the present study but studies have previously reported that eating a GF diet can be stigmatising^(^^11^^)^. Dietary habits also depend on the availability, convenience, cost and palatability of GF substitute products^(^^34^^)^. With the large increase in CD in Sweden^(^^15^^,^^38^^)^, the supply of GF products in grocery stores, restaurants and other venues is improving, possibly making it easier to adjust to the new diet. In addition, the sensory qualities of these foods are also improving, and prices are going down, which is a positive development given the usually much higher cost of GF alternatives^(^^39^^)^.

We have shown that, to a large extent, adolescents being screened for CD frequently use manufactured GF replacement products when they change to GF diet and decrease their intake of many energy-dense foods. A question that inevitably arises is whether the GF diet is healthier than the regular gluten-containing diet? Our research group will be analysing this subject in a forthcoming paper.

A question that arises from the result of the present study is whether there would have been less use of manufactured GF replacement products, and a more pronounced change in other food groups, if the families of the adolescents with CD had to pay for the products themselves. In Sweden as well as in other countries in the European Union (EU), according to EU commission regulation 41/2009, wheat starch is accepted as a GF alternative as long as gluten does not exceed 100 mg/kg product. This could result in a larger range of GF products available to Swedes with CD compared with countries outside the EU. The assortment of GF products available through the Swedish prescription system is, however, smaller than that available in ordinary grocery stores.

In Sweden the economic support that families receive from the tax-financed prescriptions of GF products is very different from the situation in many other countries where no financial support is offered. Since the prescription of GF products ends when an adolescent turns 16 years old in most county councils in Sweden, it would be interesting to examine if and how this affects the content of the GF diet and the adherence to the diet.

### Conclusions

The present results show that changing to a GF diet after a diagnosis of CD affects overall food intake. The ingredients on the plate are altered; however, this does not necessarily include a change of food groups. The intake of some popular foods are reduced but the availability of manufactured GF replacement products makes it possible for adolescents to keep many of their old food habits when diagnosed with CD.
